# Deconvolution of hippocampal RNA-sequencing data uncovers distinct cell-type-specific transcriptomic changes in depression and suicide

**DOI:** 10.1192/j.eurpsy.2025.1317

**Published:** 2025-08-26

**Authors:** Y. Dwivedi, A. Francis

**Affiliations:** 1 Psychiatry and Behavioral Neurobiology, University of Alabama at Birmingham, Birmingham, United States

## Abstract

**Introduction:**

Major depressive disorder (MDD) is a prevalent and debilitating mental health condition that affects millions of individuals globally and has the highest risk of associated suicide. The underlying etiology of depression and suicide is not clearly understood, impeding the development of effective therapy. Further, the risk factors associated with suicide among MDD patients have not been delineated.

**Objectives:**

Conducting extensive brain gene expression profiling has emerged as a valuable approach to gaining a comprehensive understanding of the root causes of MDD and MDD-associated suicide from a genomic perspective. The objective of the study was to gain further insight into the precise molecular dysregulation underlying depression and suicide at the cellular level.

**Methods:**

Transcriptome deconvolution was deployed on postmortem human hippocampus samples. The integrated dataset of the human hippocampus contained a total of 73 samples, including 29 healthy controls (C), 15 MDD non-suicide patients (D-S), and 29 MDD patients who died by suicide (D+S). Single-nucleus RNA sequencing (snRNASeq) data was analyzed to establish a reference gene expression profile for deconvolution, identifying 13 cell-type clusters encompassing 11 major hippocampal cell types. Cell type proportions were assessed using MuSic2, revealing variability across sample groups, with endothelial cells predominating, followed by pyramidal cells.

**Results:**

Cell type-specific gene expression deconvolution using bMIND showed distinct patterns among cell types and sample groups. Cell type-level differential expression analysis yielded a higher number of differentially expressed genes than the bulk transcriptome, varying across comparisons and cell types. In pyramidal neurons, a notable contrast was observed between non-suicidal and suicidal MDD patients. The former exhibited an enrichment of cytoskeleton-related pathways and molecular functions, while the latter demonstrated immune-related terms. The dysregulated genes identified as depression and suicide-specific bear relevance to the molecular implications in individuals who die by suicide due to depression.

**Conclusions:**

Our findings of cell-type-specific gene expression and functions in hippocampus serve as a foundational framework for comprehending the complexities of MDD and suicidality. The differences observed in the enriched pathways and biological processes within each cell type underscore the molecular underpinnings of the observed phenotype and delineate the risk genomic factors associated with suicide among MDD patients.

**Disclosure of Interest:**

None Declared

